# Analysis of the Mechanism of Maslinic Acid on Papillary Thyroid Carcinoma Based on RNA-Seq Technology

**DOI:** 10.1155/2022/7000531

**Published:** 2022-09-07

**Authors:** Rong Li, Yanjiao Zhang, Runqing Xiang, Aihe Lin, Zongxiao Xia, Xiaomei Long, Shuang Guo, Yuan Fan, Zukun Chen

**Affiliations:** ^1^Yunnan University of Chinese Medicine, Kunming 650500, Yunnan, China; ^2^Yunnan Union of Medicinal Herbs Cultivation, Kunming 650500, Yunnan, China; ^3^Haiyuan College, Kunming Medical University, Kunming 651700, Yunnan, China; ^4^The Second Affiliated Hospital of Yunnan University of Chinese Medicine, Kunming 650216, Yunnan, China

## Abstract

**Objective:**

This study analyzed gene sequence changes in the thyroid papillary carcinoma (PTC) cell line TPC-1 treated with the natural compound maslinic acid (MA) through RNA-sequencing (RNA-seq) and identified the necessary genes to provide a basis for the study of the molecular mechanism of action of MA in PTC treatment.

**Methods:**

RNA-seq technology was used to detect genetic differences between the normal cell group (Nthy-ori 3-1) and the TPC-1 cell group (N vs T). Then, gene ontology (GO) analysis, Kyoto Encyclopedia of Genes and Genomes (KEGG) analysis, Venn diagram analysis of shared genes, and protein–protein interaction (PPI) network analysis were used to analyze the therapeutic effect of the MA on TPC-1 cells. Real-time quantitative PCR (qRT-PCR) was used to verify six key genes.

**Results:**

GO and KEGG analyses showed that four crucial signaling pathways are related to TPC development: cytoplasmic molecule (cell adhesion molecules), neuroactive ligand–receptor interaction, tumor transcriptional disorder, and cytokine–cytokine interaction. The Venn diagram revealed 434 genes were shared between the MA vs T-group and 387 genes were shared between the MATH vs *T* and N vs *T* groups. PPI and ClueGO showed that NLRP3, SERPINE1, CD74, EDN1, HMOX1, and CXCL1 genes were significantly associated with PTC, while CXCL1, HMOX1, and other factors were mainly involved in the cytokine–cytokine interaction. The qRT-PCR results showed that the expression of NLRP3, EDN1, HMOX1, and CXCL1 genes was significantly upregulated in the TPC-1 group but significantly downregulated after MA treatment (*p* < 0.01). SERPINE1 and CD74 genes were not expressed in TPC-1 cells, whereas they were significantly upregulated after MA treatment (*p* < 0.01).

**Conclusions:**

This present study proves for the first time that MA can treat PTC, and the preliminary identification of key genes and rich signal transduction pathways provides potential biomarkers. It also provides potential biomarkers for the treatment of PTC with the natural compound MA and preliminarily discusses the therapeutic mechanism of action of MA against PTC, which is helpful for the further diagnosis and treatment of PTC patients.

## 1. Introduction

Thyroid carcinoma is the most common thyroid malignancy. According to the pathological type, it is mainly divided into five types: follicular thyroid carcinoma, papillary thyroid carcinoma (PTC), thyroid medullary carcinoma, anaplastic thyroid carcinoma, and undifferentiated carcinoma. PTC is the most common thyroid cancer that belongs to differentiated thyroid cancer, accounting for approximately 88% of thyroid cancer. The incidence rate of PTC is increasing year by year, mostly accompanied by lymph node metastasis [1]. Modern related research shows that the PTC is involves multiple genes and targets. Researchers have usually found that the TPC-1 pathogenesis is associated with sseveral classical pathways, including *BRAF* mutation, RET/PTC rearrangement [2], PI3K/AKT/mTOR [3, 4], and MAPK/ERK [5].

MA is a common pentacyclic triterpene compound mainly found in olive fruits and leaves [6, 7], pomegranate flowers [8], and other natural plants. MA has various pharmacological activities, including antitumor [9], anti-AIDS [10], antioxidant [11], and antidiabetes [12]. Through the PI3K/AKT/NF-*κ*B signaling pathway, MA plays an anti-inflammatory role [13]. MA inhibits the growth of neuroblastoma cells by inducing apoptosis, activating caspase, inhibiting cell migration and invasion, and targeting the MAPK/ERK signaling pathway [14]. It exhibits an inhibitory effect on precancerous colon lesions in rats [15]. MA can significantly inhibit tumor formation in azomethane/dextran sodium sulfate mice and xenotransplantation mice and regulate the AMPK/mTOR pathway, thereby inhibiting the growth of colon tumors [16]. In addition, MA can induce apoptosis of pancreatic cancer cells [17] and act against breast cancer [18].

Gene sequencing technology can obtain the gene information of each sample through sample RNA extraction, primer design, sequencing, and bioinformatics sequence analysis, and can reflect the expression and regulation of genes in cells as a whole. Gene sequencing has become the most commonly used technology for studying the mechanisms of modern molecular pharmacology. It can widely and deeply mine the molecular genes of diseases to identify key target genes. Next-generation gene sequencing showed that the polycystic kidney disease and liver disease like protein 1 (PKHD1L1) may be a tumor suppressor gene in thyroid cancer [19]. Genomic DNA sequencing and mutation detection of PTC tumors showed that VEGF expression in PTC tissues was significantly lower than that in adjacent normal thyroid tissues, which was significantly related to invasive tumor behaviors such as lymph node metastasis and mutation [20]. Numerous studies on PTC have been conducted using next-generation gene sequencing. Zheng et al. [19] found significant differences in the expression of the PKHD1L1 gene between thyroid carcinoma and adjacent healthy thyroid tissues through next-generation sequencing. Yin et al. [20] detected *BRAF* mutations in PTC tumor tissues through genomic DNA sequencing. The results showed that CCDC67 expression was downregulated in PTC, and this expression was negatively correlated with PTC invasiveness and *BRAF* mutations.

Currently, no study has been reported on the role of the MA in PTC treatment. In this study, RNA-seq was used to detect TPC-1 cells and MA-treated TPC-1 cells to analyze the changes in the molecular function and signal transduction pathway and thus find target genes, offer broader information for the inhibition of TPC-1 proliferation, and provide more evidence for MA to treat PTC.

## 2. Materials and Methods

### 2.1. Cell Lines and Culture Conditions

The human PTC cell line TPC-1 and human normal thyroid cells (Nthy-ori3-1) cells were purchased from the American Type Culture Bank (USA). Both cells were cultured in RPMI/1640 medium containing 10% fetal bovine serum (Gibco, USA) and 1% penicillin/streptomycin at 37°C in a 5% CO_2_ incubator. The medium was changed regularly. The cells were divided into Nthy-ori3-1, TPC-1, and MA + TPC-1 cell groups. MA (purity>98%) was purchased from Sichuan Weikeqi Biological Technology Co., Ltd., China.

### 2.2. RNA Isolation and Sequencing

Nthy-ori3-1 and TPC-1 cells were cultured in RPMI/1640 medium for 24 h, and TPC-1 cells (66,700 cells/mL) were cultured in the medium containing 12, 24, and 48 *μ*MMA for 48 h. The cells were washed with PBS and lysed using the TRIzol reagent (MRC, Germany). The RNA concentration (OD260/280 ratio) was detected using a nano-photometer spectrophotometer, and the RNA integrity was accurately detected using the Agilent 2100 Bioanalyzer (Agilent Technologies, USA). After passing the quality inspection, different libraries were pooled according to the effective concentration and target offline data volume and then sequenced using Illumina, and a 150-bp paired-end read was generated. Four fluorescently labeled dNTPs, DNA polymerase, and adaptor primers were added to the sequenced flow cell for amplification. When each sequencing cluster extends the complementary strand, each fluorescently labeled dNTP releases the corresponding fluorescence. The sequencer then captures the fluorescent light signal and converts it into a sequencing peak through computer software, thereby obtaining the sequence information of the fragment to be tested.

### 2.3. Differential Gene Expression analysis

Differentially expressed genes (DEGs) are genes whose expression levels have changed significantly. The gene expression differences between the *T*  vs N, MATL vs *T*, MATM vs *T*, and MATH vs *T* groups were calculated. The DEGs were filtered and analyzed according to the following criteria: condition setting (|Fold change| > 2, *p* < 0.05).

### 2.4. Gene Ontology and Kyoto Encyclopedia of Genes and Genomes Analyses

Volcano map and heat map analyses were performed on the four groups of DEGs to determine the changes in genes and enrichment of upregulated and downregulated genes. In the enrichment analysis of *T* vs N and MA vs *T* groups, Gene Ontology (GO) and Kyoto Encyclopedia of Genes and Genomes (KEGG) were used for DEG analysis. The first 20 enrichment pathways and the first 10 meaningful functional genes were selected, and their similarities were analyzed.

### 2.5. Data Visualization Analysis to Identify Essential Gene Targets

The Venn diagram was used to determine the shared genes of MATH, MATM, MATL vs *T*, and *T* vs N groups, and the Java plug-in ClueGo in Cytoscape 3.8.0 (http://www.cytoscape.org) was used to compare the shared genes between MATH vs *T*, MATM vs *T*, MATL vs *T*, and MA administration groups and the T vs N group for enrichment analysis. In addition, the protein interaction (PPI) network analysis was performed using string (https://string-db.org/) to enrich the genes. The protein node map was imported into Cytoscape 3.8.0, data were visualized, key target genes were identified, degree values were combined, and gene enrichment was performed in ClueGO (showed genes that are enriched) and then the key genes were verified.

### 2.6. Verification of the Expression of Related Factors through qRT-PCR

After sequencing the same batch of RNA samples, total RNA quantification and reverse transcription were performed to synthesize cDNA, and the 2 × Power Up SYBR Green Master Mix kit was used to verify key genes according to the primer sequences listed in Table 1.

### 2.7. Data Analysis

The SPSS 23.0 software was used for repeated measurement analysis of variance (ANOVA). Differences between groups were analyzed using the LSD test and one-way ANOVA. If *p* < 0.05, the difference was considered significant. In addition, GraphPad Prism 8 (GraphPad Software, Inc.) was used to draw each group of bar graphs.

## 3. Results

### 3.1. N vs T and MA vs T differential expression analysis

**Figure 1:**
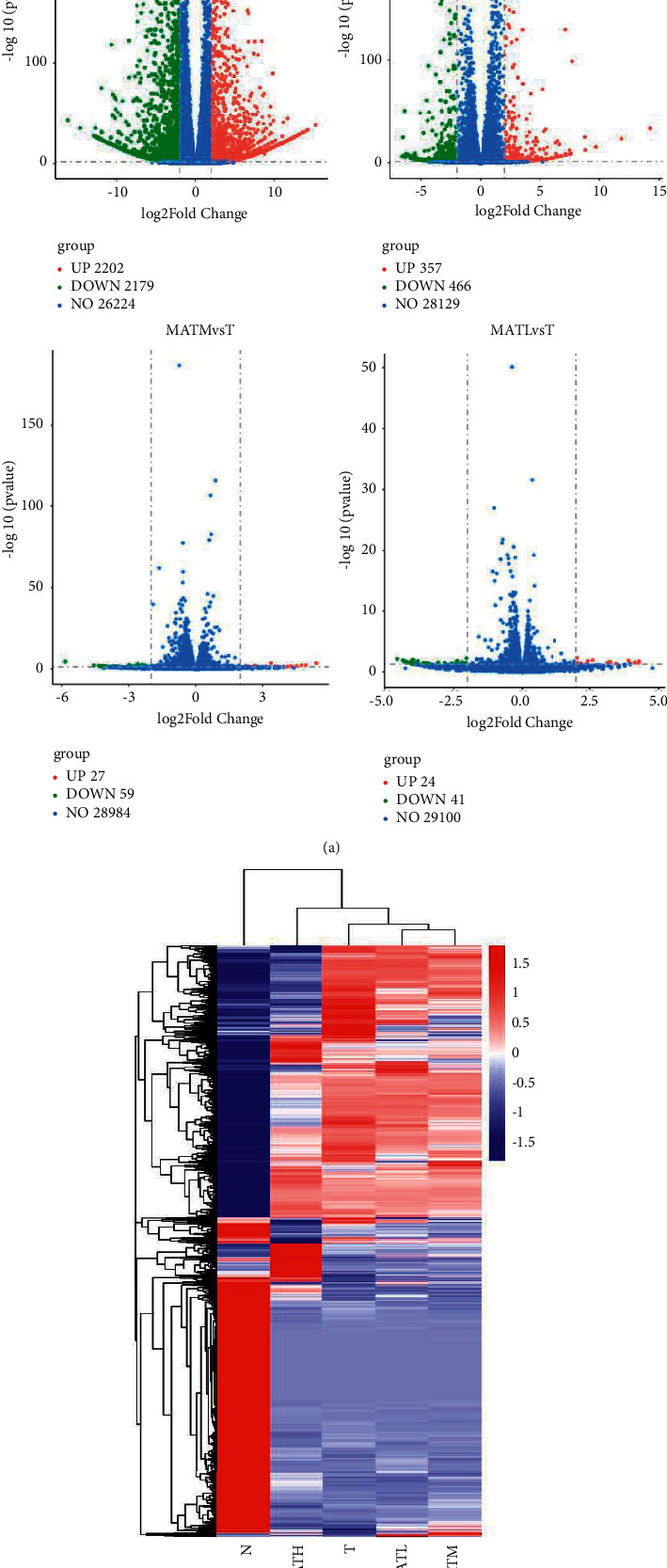
Gene volcano map (a). Cluster analysis of differential genes (b). In the volcano map, blue dots indicate that genes have no significant differences, red origin indicates that genes are upregulated, and green origin indicates that genes are downregulated. In the MA vs T heatmap analysis, each row or column of the heatmap represents a module. The darker the color of the module, the more red or blue, the higher the correlation.

According to the conditions of |Fold change| > 2 and *p* < 0.05 for the screening of genes, N vs *T* gene volcano map exhibited 4381 differential genes. Of these genes, 2202 were upregulated and 2179 were downregulated. The MATH vs *T* group exhibited 823 different genes, including 357 upregulated and 466 downregulated genes. The MATM vs *T* group exhibited 86 different genes including 27 upregulated and 59 downregulated genes The MATL vs *T* group had 65 different genes. Of them, 24 genes were upregulated and 41 were downregulated (Figure 1(a)). In addition, the MATH group was more similar to the *T* group (Figure 1(b)).

### 3.2. GO and KEGG Analyses in the *T* vs N and MA vs *T* Groups

**Figure 2:**
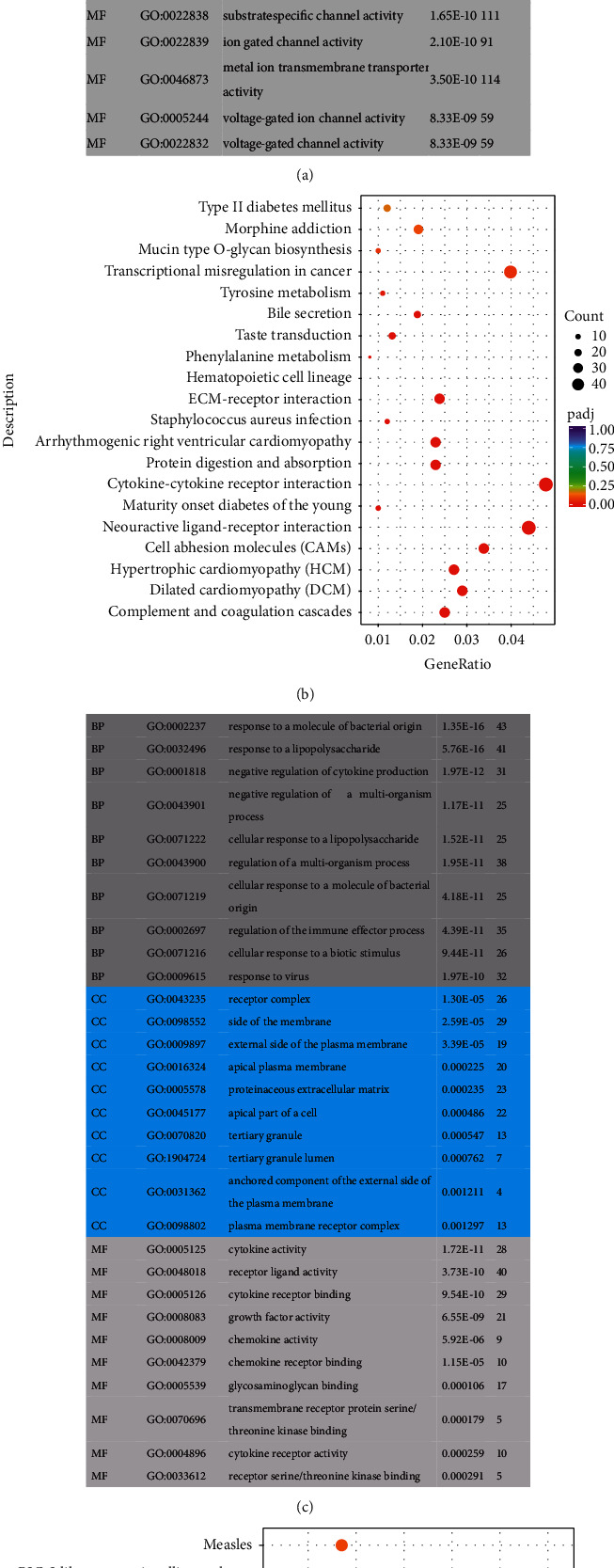
(a) GO analysis revealed the top 10 significantly enriched terms of the three major functions for the T vs N group; (b) KEGG analysis revealed the top 20 most significant genes and genomic pathways in this group; (c) GO analysis revealed the top 10 significantly enriched terms of the three major functions for the MA vs T group; G: KEGG analysis revealed the top 20 most significant genes and genomic pathways in this group.

GO analysis found that the selected DEGs in the *T*  vs N group were mainly involved in biological processes such as extracellular matrix organization, extracellular structure organization, and neuron projection guidance, and cell composition, including extracellular matrix, proteinaceous extracellular matrix, and receptor complex. In biological processes, cell composition and molecular function are the most common functions. According to KEGG analysis, genes and genomic pathways related to the cytokine–cytokine receptor interaction, neuroactive ligand–receptor interaction, transcriptional misregulation in cancer, cell adhesion molecules, etc. were significantly enriched in the *T*  vs N group (Figures 2(a) and 2(b)). The MA vs *T* group mainly participated in biological processes, such as responding to a molecule of bacterial origin, a response to a lipopolysaccharide, and negative regulation of cytokine production; cell composition, including the receptor complex, side of the membrane, and external side of the plasma membrane; and molecular functions such as cytokine activity, receptor ligand activity, and cytokine receptor binding. According to the KEGG analysis, pathways mainly enriched in the MA vs *T* group were the cytokine–cytokine receptor interaction, the IL-17 signaling pathway, and the TNF signaling pathway (Figures 2(c) and 2(d)). The cytokine receptor interaction pathway was significantly enriched in the *T* vs N and MA vs *T* groups.

### 3.3. Analysis  of  DEGs shared by the MA vs *T*  and *T* vs N Groups

**Figure 3:**
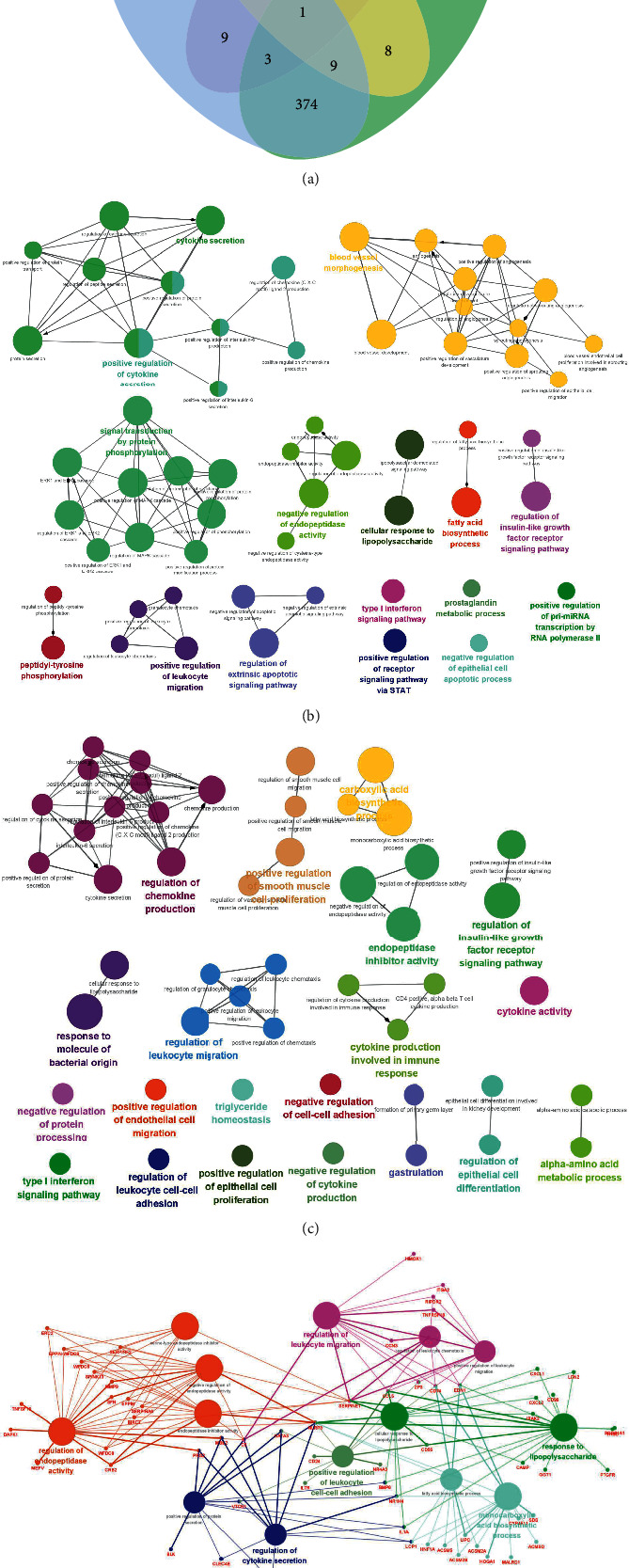
(a) Venn diagram analysis of common genes of each group; (b) use of ClueGO in Cytoscape for analyzing the enrichment of MATH, TM, and TL vs T groups; c and d: analysis of common genes to determine the key genes.

Venn diagram analysis of MATH, *M*, L vs *T*, and *T* vs N groups revealed that 434 genes were shared between the MA vs T and T vs N groups. In total, 15 genes were shared between the MATL vs T and N vs T groups, 32 genes were shared between the MATM vs T and N vs *T* groups, and 387 genes were shared between the MATH vs *T* and N vs *T* groups (Figure 3(a)); the total gene PPI (TSV format) was calculated. The degree values are shown in Table 2. In addition, the analysis of MATL vs *T*, MATM vs *T*, and MATH vs *T* groups by using ClueGO revealed that the MATH vs *T* group was most correlated with the N vs *T* group (Figure 3(b)). An analysis of the shared genes of the MA vs *T*  and *T*  vs N groups showed that their main molecular functions were related to cytokine secretion; this result is basically the same as that of the previous analysis (Figure 3(c)).We then visually analyzed the data, combined with the calculation of the shared gene degree values, and found that the genes with the highest correlation were mainly SERPINE1, NLRP3, CD74, and EDN1 (Figure 3(d); Table 2).

Table 2. The PPI analysis results were imported into the Cytoscape to calculate the degree value, and the genes with a degree value of ≥4 were selected. MMP9, matrix metallopeptidase 9; CCL5, C-C motif chemokine ligand 5; CXCL1, C-X-C motif chemokine ligand 1; SERPINE1, serpin family *E* Member 1; NLRP3, NLR family pyrin domain containing 3; KRT5, Keratin 5; THBD, thrombomodulin; HMOX1, heme oxygenase 1; BMP2, bone morphogenetic protein 2; IL1A, interleukin 1 Alpha; MX2, MX dynamin-like GT Pase 2; EDN1, endothelin 1; CD24, CD24 molecule; CD55, CD55 molecule; IL7R, interleukin 1 receptor; SERPINF2, serpin family F member 2; GSTA1, glutathione S-transferase alpha 1; CD74, CD74 molecule. Source: Genecards (https://www.genecards.org/).

### 3.4. qRT-PCR Verifies the Expression of Related Genes

**Figure 4:**
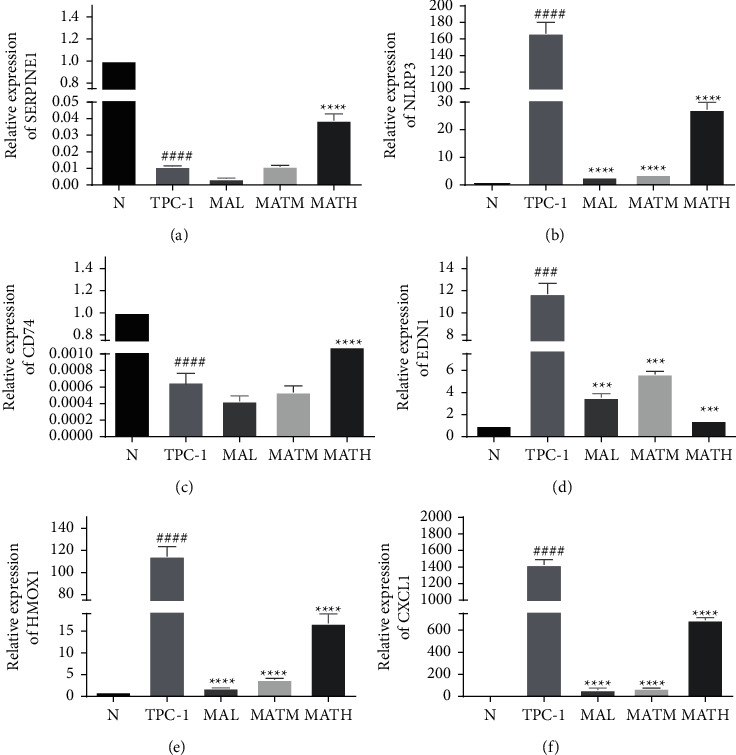
Histograms of qRT-PCR results. (a) b, (c) d, (e), and f represent the relative expression of SERPINE1, NLRP3, CD74, EDN1, HMOX1, and CXCL1 genes respectively, *p* < 0.01.

SERPINE1, NLRP3, CD74, EDN1, HMOX1, and CXCL1 were verified as the key genes through qRT-PCR. The results showed that compared with normal cells (N), the NLRP3, EDN1, HMOX1, and CXCL1 genes were upregulated in the cancer cell group (*T*). Compared with the *T* group, the MATH group among the administration groups showed a downregulation trend (*p* < 0.01). However, the downregulation of NLRP3, HMOX1, and CXCL1 genes in the MATH group was significantly lower than that in the MATL and MATM groups (Figures 4(b), 4(d), 4(e), 4(f)); downregulated genes are SERPINE1 and EDN1, with significant differences (*p* < 0.01) (Figures 4(a) and 4(c)), and the MATH group was significantly upregulated (*p* < 0.01) (Figures 4(b), 4(d), 4(e), 4(f)).

## 4. Discussion

MA is a naturally occurring pentacyclic triterpene compound. Being an excellent pharmacological active product, MA has attracted people's interest because of its high-quality biological properties. MA has an inhibitory effect on various tumors. It targets the MAPK/ERK signaling pathway and inhibits cell migration and invasion. As the MA concentration increases, the inhibition rate becomes stronger [14]. MA can also inhibit the proliferation of renal cancer cell lines and the angiogenesis of endothelial cells, thereby exhibiting an antirenal cancer effect [21]. It can also prevent the formation of colon tumors by inhibiting the AMPK/mTOR signaling pathway [22]. MA exhibits numerous biological activities. However, researchers are yet to unravel how MA therapeutically targets oncogenic cell signaling cascades in different cancers [23]. Moreover, the effect of MA on PTC and its mechanism of action require further study.

The sequencing results showed that TPC-1 DEGs were significantly higher in the presence of MA, with the number of downregulated genes being significantly higher than that of upregulated genes. This suggests that MA mainly acts as an inhibitor of TPC-1 cells. The results of GO analysis of MA action on TPC-1 cells showed that MA and *T* groups were mainly involved in biological processes such as bacterial-derived molecular reactions, lipopolysaccharide reactions, and negative regulation of cytokine production. Cell composition analysis revealed correlations between the receptor complex side of the membrane, the outer side of the plasma membrane, cytokine activity, receptor ligand activity, and cytokine receptor binding. According to the KEGG analysis, the pathways mainly enriched were the cytokine–cytokine receptor interaction, the IL-17 signaling pathway, and the TNF signaling pathway, with the cytokine–cytokine receptor interaction significantly enriched in the *T* vs N and MA vs T-groups. ClueGO analysis of MA action on TPC-1 revealed that the correlation between the MATH vs *T*, MATM vs *T*, and MATH vs *T* groups was the highest. Analysis of shared genes in the MAvs *T* and T vs N groups revealed that their main molecular functions were related to cytokine secretion. This result is essentially the same as that of the previous analysis (Figure 3(c)). Then, visual analysis of the data combined with calculation of the shared gene degree values, revealed that the SERPINE1, NLRP3, CD74, and EDN1 genes were closely related to these genes.

In the present study, the bioinformatics function of the MA on TPC-1 cells was analyzed through next-generation gene sequencing. MA induced TPC-1 cells by triggering cytokine receptor interaction signals and through regulation of various cytokine functions. At 48 *μ*MMA, the most common genes were shared with TPC-1 cells. Moreover, SERPINE1, NLRP3, CD74, EDN1, HMOX1, and CXCL1 were verified as the key genes by qRT-PCR. The results were consistent with those of gene sequencing. Importantly, gene enrichment analysis revealed that HMOX1, CXCL1, NLRP3, and other genes were found to be involved in the cytokine signaling pathway and jointly promote the anticancer effect of MA. Gene sequencing enables us to better understand the molecular mechanisms of the anti-TPC-1 effect of MA and provides a new clue for the further study of its pharmacological activity.

## Figures and Tables

**Table 1:** Gene primer sequences.

Gene	Primer sequence	Amplified product length
SERPINE1	Forward, 5′-CTCATCAGCCACTGGAAAGGCA-3′	154bp
	Reverse, 5′-GACTCGTGAAGTCAGCCTGAAAC-3′	

NLRP3	Forward, 5′-GGACTGAAGCACCTGTTGTGCA-3′	153bp
	Reverse, 5′-TCCTGAGTCTCCCAAGGCATTC-3′	

CD74	Forward, 5′-AAGCCTGTGAGCAAGATGCGCA-3′	134bp
	Reverse, 5′-AGCAGGTGCATCACATGGTCCT-3′	

EDN1	Forward, 5′-CTACTTCTGCCACCTGGACATC-3′	126bp
	Reverse, 5′-TCACGGTCTGTTGCCTTTGTGG-3′	

CXCL1	Forward, 5′-AGCTTGCCTCAATCCTGCATCC-3′	119bp
	Reverse, 5′-TCCTTCAGGAACAGCCACCAGT-3′	

HMOX1	Forward, 5′-CCAGGCAGAGAATGCTGAGTTC-3′	144bp
	Reverse, 5′-AAGACTGGGCTCTCCTTGTTGC-3′	

*β*-actin	Forward, 5′-CACCATTGGCAATGAGCGGTTC-3′	250bp
	Reverse, 5′-AGGTCTTTGCGGATGTCCACGT-3′	

**Table 2:** Degree values of genes shared between the MA vs T and T vs N groups.

Nodes	Degree	Nodes	Degree
MMP9	20	IL1A	8
CCL5	18	MX2	8
CXCL1	15	EDN1	8
SERPINE1	14	CD24	7
NLRP3	12	CD55	6
KRT5	12	IL7R	6
THBD	12	SERPINF2	5
HMOX1	11	GSTA1	5
BMP2	9	CD74	4

## Data Availability

The data used to support the findings of this study are included within the article.
